# Exploring spatial patterns, and identifying factors associated with insufficient cash or food received from a productive safety net program among eligible households in Ethiopia: a spatial and multilevel analysis as an input for international food aid programmers

**DOI:** 10.1186/s12889-023-16001-2

**Published:** 2023-06-14

**Authors:** Addisalem Workie Demsash, Milkias Dugassa Emanu, Agmasie Damtew Walle

**Affiliations:** 1 Mattu University, College of Health Science, Health Informatics Department, Mettu, Ethiopia; 2 Mattu University, College of Health Science, Nursing department, Mettu, Ethiopia

**Keywords:** Cash, Food, Safety net program, Households, And spatial patterns

## Abstract

**Background:**

In low-income countries, households’ food insecurity and the undernutrition of children are the main health problems. Ethiopia is vulnerable to food insecurity and undernutrition among children because its agricultural production system is traditional. Hence, the productive safety net program (PSNP) is implemented as a social protection system to combat food insecurity and enhance agricultural productivity by providing cash or food assistance to eligible households. So, this study aimed to explore spatial patterns of households’ insufficient cash or food receiving from PSNP, and identify its associated factors in Ethiopia.

**Methods:**

The 2019 Ethiopian Mini Demographic and Health Survey dataset was used. A total of 8595 households were included in this study. Data management and descriptive analysis were done using STATA version 15 software and Microsoft Office Excel. ArcMap version 10.7 software was used for spatial exploration and visualization. SaTScan version 9.5 software was used for spatial scan statistics reports. In the multilevel mixed effect logistic regression analysis, explanatory variables with a *p*-value of less than 0.05 were considered significant factors.

**Results:**

Overall, 13.5% (95% CI: 12.81–14.27%) of the households’ level beneficiaries received cash or food from PSNP. The spatial distribution of households’ benficiaries received cash or food from PSNP was not random, and good access to cash or food from PSNP was detected in Addis Ababa, SNNPR, Amhara, and Oromia regions. Households’ heads aged 25–34 (AOR:1.43, 95% CI: 1.02, 2.00), 35–44 (AOR: 2.41, 95% CI: 1.72, 3.37), and > 34 (AOR: 2.54, 95% CI: 1.83, 3.51) years, being female (AOR: 1.51, 95% CI: 1.27,1.79), poor households (AOR: 1.91, 95% CI:1.52, 2.39), Amhara (AOR:.14, 95% CI: .06, .39) and Oromia (AOR:.36, 95% CI:.12, 0.91) regions, being rural residents (AOR:2.18, 95% CI: 1.21,3.94), and enrollment in CBHS (AOR: 3.34, 95% CI:2.69,4.16) are statistically significant factors.

**Conclusions:**

Households have limited access to cash or food from the PSNP. Households in Addis Ababa, SNNPR, Amhara, and Oromia regions are more likely to receive benefits from PSNP. Encouraging poor and rural households to receive benefits from the PSNP and raise awareness among beneficiaries to use the benefits they received for productivity purposes. Stakeholders would ensure the eligibility criteria and pay close attention to the hotspot areas.

## Background

Household food insecurity occurs when households lack adequate physical, social, and economic access to adequate and nutritious food to meet their members’ nutrition requirements for an active and healthy lifestyle [1, 2]. Households’ food insecurity is one of the underlying causes of all types of malnutrition, including insufficient quantity, poor quality, and diet inconsistency [3, 4]. So, it is a significant problem to achieve international nutritional goals for children. For instance, according to the United Nations International Children’s Emergency Fund (UNICEF) report, the proportion of children eating a diverse diet is around 33.33%, and the world’s poorest children would eat 20% by the year 2018 [5]. According to the Global Nutrition Report, 51% of children around the world get the recommended minimum number of daily meals, and only 16% of children eat a minimum acceptable diet [6].

Household food insecurity and the undernutrition of children are the main health problems in the world. In Africa, 52.5% of the population suffered from moderate or severe food insecurity, and the level of food insecurity in Sub-Saharan and Eastern Africa is 57.7% and 62.7%, respectively [7]. Children in low-income countries have been faced with nutritional-related problems. For instance, 20% and 7.5% of under-five children were stunted and suffered from wasting, respectively [8]. In Ethiopia, 77.1% of households suffered from food insecurity [4], and 37% and 7% of under-five children were stunted and wasted, respectively [9].

To overcome food insecurity and nutritional health-related problems, a rapid social protection agenda has been implemented as a solution or emergency support with the delivery of predictable cash transfers, food support, or both to large numbers of food-insecure people and households [10, 11]. The PSNP in Ethiopia was launched in 2015 with the support of development partners as a mechanism to respond to food insecurity by targeting five million chronically food insecure people to smooth food consumption, strengthen households' asset building, and build resilience to shocks [12].

In areas where households are dependent on agriculture for their livelihood, and have low access to technology for farming, food insecurity, and child malnutrition remain an issue. So, PSNP has a positive nutritional impact on children and food-insecure households [13]. The program also works to overcome poverty by creating assets for poor households, allowing them to use their assets for production [14], improving access to services and natural resources, and  by rehabilitating and enhancing natural environments [13].

Although Ethiopia is struggling to achieve food self-sufficiency, food production in the country is heavily constrained by human-made and natural disasters, and outdated agricultural production technologies [15]. Accordingly, the country needs social support from partners to achieve food security and agricultural productivity [16]. Therefore, the PSNP offers cash or food to promote food access, household asset creation, human capital development, enhanced agricultural productivity, and technology adoption [17].

The program first identifies households that are chronically food insecure and then provides a minimum of five days of payment per month for six months when there is a low agricultural production season for at least the next five years [18]. The program transfers food (15 kg of grain), cash (2.8 US dollars), or both to targeted households per month to smooth their food consumption and close the gap created by the annual food shortage [19]. It also makes adjustments to the wage rates over the period of the program when there is high inflation [20]. In 2008, nearly 250 thousand beneficiary households benefited from PSNP in the Bale Zone of Ethiopia, and almost 500 thousand poor households benefited from PSNP between 2008 and 2012 in Ethiopia [21]. In southern Ethiopia, one-half (49.37%) of households are beneficiaries of the program [22].

This shows that the program is bringing considerable numbers of poor households out of poverty.

However, while the PSNP has a great impact on food security and asset building [18, 20], the program also develops a sense of dependency syndrome [23]. The PSNP was not effective in reducing the rate of poverty in Ethiopia because the poverty rate in Ethiopia in 2015 was 90.20% which is only a 5.6% decline from 2004 [7]. In Ethiopia, many households are unable to afford health care costs, which is impairing their health status. Poor households are still borrowing and selling their assets to meet their healthcare expenses, and this leads them to poverty, which further makes them unable to break the economic hardship [24]. The program was not widely expanded or implemented across the regions of Ethiopia; however, the population of the country, especially the rural population, is equally vulnerable to poor agricultural production and technology utilization. Since 1998, Ethiopia's annual food aid recipients have ranged from 5 to 14 million [25]. The status of food insecurity among beneficiary households and the nutritional status of children are still severe health problems in Ethiopia. The program’s implementation is significantly affected by age, the educational status of the household head, the occurrence of shock, a lack of monitoring and unsustainable management, a lack of logistical support, limited payment, and the beneficiary’s awareness level [21]. So, an assessment of the implementation of PSNP is necessary.

There have been no spatial studies on how many households receive PSNP benefits. Additionally, spatial studies have not been carried out to determine in which areas of the country the program was practically implemented. Spatial studies are important for determining and making decisions about which areas of Ethiopian households received and did not receive PSNP benefits without disclosing their confidential information. Because they can specifically show the issues through a map, spatial studies have implications for the programmer's ability to make decisions effectively and efficiently without delay or resource waste. Research on the program’s implementation is required to address this knowledge gap, which further supports programmers’ efforts to evaluate, ensure the households’ selection criteria, and redesign. Therefore, this study aimed to explore spatial patterns and point out factors associated with cash or food received from the PSNP among eligible households in Ethiopia.

## Methods

### Study design and setting

The cross-sectional study design was conducted across the region of Ethiopia. Ethiopia is located in the Horn of Africa and bordered by Eritrea to the north, Djibouti, and Somalia to the east, Sudan and South Sudan to the west, and Kenya to the south. Ethiopia has nine regional states with two administrative cities. These are subdivided into different administrative units (68 zones, 817 woredas, and 16,253 kebeles). Ethiopia has a population of 105 million [26], and its economy is depend on agriculture, which accounts for 40% of the growth development plan. and improving livelihoods and nutrition can become a long-lasting solution to Ethiopia’s chronic poverty and food insecurity [27].

### Data source

For this study, the 2019 Ethiopian Mini Demographic and Health Survey (EMDHS) dataset was used from the Demographic and Health Survey (DHS) program website (https://dhsprogram.com/Data/terms-of-use.cfm). The 2019 EMDHS data represents Ethiopia’s second DHS. The Ethiopian Federal Ministry of Health (EFMH) requested the Ethiopian Public Health Institute (EPHI) to implement the survey. The survey was conducted with the financial and technical support of the World Bank, UNICEF, and the United States Agency for International Development (USAID). The survey was conducted by EPHI in collaboration with the Central Statistical Agency (CSA) from March 21 to June 28, 2019. The 2019 EMDHS generates data for measuring the progress of the health sector goals set under the Growth and Transformation Plan (GTP), which is closely aligned with the Sustainable Development Goals (SDG) [9]. The Ethiopian geographical shapefiles were downloaded from the Open Africa website (https://africaopendata.org/dataset/ethiopia-shapefiles).

### Sampling techniques and study population

The sampling frame used for the 2019 EMDHS is a frame of all census enumeration areas (EAs) created for the 2019 Ethiopia Population and Housing Census (EPHC) and conducted by the Central Statistical Agency (CSA). The census frame is a complete list of the 149,093 EAs, covering an average of 131 households, created for the 2019 EPHC. The sample for the 2019 EMDHS was designed to provide an estimation of key indicators for the country as a whole, for urban and rural areas separately, and for each of the nine regions and the two administrative cities.

Two-stage stratified cluster sampling was used. Each region was stratified into urban and rural areas. In the selected EAs, a household listing operation was done, and the results were used as a sampling frame for household selection in the second stage. Finally, a fixed number of households per cluster were selected. Samples of EAs were selected independently in each stratum through implicit stratification and equal proportional allocation. Standard questionnaires were adapted to reflect the population and health issues relevant to Ethiopia and donors. The household questionnaire was one of the questionnaires used to list all of the usual members of and visitors to the selected households. Basic information was collected from each listed person. The data on age and sex were used to identify eligible women for individual interviews. So, these women were the source population. Whereas, all eligible women (aged 15–49) who were either permanent residents of the selected households or visitors who slept in the household the night before the survey were the study population. Details about the methods of the survey are available from EMDHS [9].

### Study variables

#### Independent variables

Socio-demographic characteristics of households such as wealth status, age, sex, and media access (Television, Radio) were considered as individual-level independent variables, whereas the place of residency and Region were extracted as community-level independent variables.

#### Dependent variable

The dependent variable of the study was the receiving cash or food from the productive safety net program.

#### Operational definition

### Media exposure

If the households had either radio or television or both, then the households were exposed to media; otherwise, they were not exposed to media [28].

Receiving cash or food from a productive safety net program.

A social protection system is being implemented in Ethiopia. The program targets chronically food-insecure households by providing cash or food to the beneficiaries regularly, either for work, free, or both [4, 25]. So, if the households received sufficient cash or food directly from the PSNP in the regular period (five years), it is considered yes; Otherwise no.

### Data management and statically analysis

Data cleaning was performed to prepare the data for analysis according to the objectives of the study. Variables were recoded to meet the desired classification. To ensure the representativeness of survey results at the national level [29], sampling weights were applied during the analysis. The STATA version 15 software and Microsoft Office Excel were used for data management and statistical analysis.

### Spatial data analysis

ArcMap version 10.7 software was used for spatial autocorrelation and detection, as well as for the interpolations of households receiving cash, or food from the PSNP in Ethiopia.

### Global spatial autocorrelation

The global spatial autocorrelation (Global Moran’s I) statistic measure was used to assess whether receiving cash or food from the PSNP was dispersed, clustered, or randomly distributed in Ethiopia [30]. Moran’s I value is close to -1, close to + 1, and zero (**0**), indicating a dispersed, clustered pattern, and random distribution of households receiving sufficient cash or food from the PSNP, respectively [31, 32]. The z scores and *p*-values were used to determine whether receiving cash or food from the PSNP in Ethiopia is a hot spot or cold spot in the spatial SaTScan analysis.

### Spatial interpolation

Unsampled areas were predicted by the spatial interpolation of receiving cash or food from the PSNP based on sampled EAs. For the prediction of unsampled EAs, the Kriging Gaussian interpolation technique were used.

### Spatial scan statistics

Sat Scan version 9.5 software was used for the local cluster detection analysis [33]. We employed purely spatial Bernoulli-based model scan statistics to determine the geographical locations of statistically significant clusters with high rates of cash, or food received from the PSNP among households [34]. Those households that did not receive cash or food from the PSNP were taken as cases, and those that receive cash or food from the PSNP were taken as controls to fit the purely spatial Bernoulli model for the scanning window that moves across the study area. The scanning window that moved outside the study area was clipped. The default maximum spatial cluster size of less than 50% of the population was used as an upper limit, allowing both small and large clusters to be detected, and ignoring clusters that contained more than the maximum limit because of the circular shape of the window. For each potential cluster, a log-likelihood ratio test statistic was used to determine if the number of observed cases within the cluster was significantly higher than expected or not. The circle with the maximum likelihood ratio test statistic was defined as the most likely cluster, then compared with the overall distribution of maximum values. All significant clusters were identified, assigned *p* values, and ranked based on their likelihood ratio test based on the 9999 Monte Carlo replications [35].

### Multilevel logistic regression analysis

Since the data source had a hierarchical nature, the assumption of independence and equal allocation would be violated. The authors assumed that a multilevel mixed-effect logistic regression model was best to overcome the dependency between clusters and correlations between respondents. So, a multilevel mixed-effect logistic regression analysis was employed. To alleviate dependency and correlations between the records, we assumed four models, such as model A (a null model that assesses the households receiving cash or food from PSNP), model B (countian individual-level variables), model C (countian community-level variables), and model D (the aggregate model of models 2 and 3). The overall multilevel model of the households’ probability of receiving cash or food from PSNP was described as follows [36, 37]:

$${\varvec{L}}\mathbf{o}\mathbf{g}\left(\frac{{\varvec{\uppi}}\mathbf{i}\mathbf{j}}{1-{\varvec{\uppi}}\mathbf{i}\mathbf{j}}\right)={\varvec{\upbeta}}0+{\varvec{\upbeta}}1\mathbf{X}\mathbf{i}\mathbf{j}+{\varvec{\upbeta}}2\mathbf{Z}\mathbf{i}\mathbf{j}+{\varvec{\upmu}}\mathbf{j}+\mathbf{e}\mathbf{i}\mathbf{j}$$

where **i** and **j** are individual and community-level units, respectively; **X** and **Z** refer to individual and community-level variables, respectively; **πij** is the households’ probability of receiving cash or food from PSNP for the **i**^**th**^ households’ respondent in the** j**^**th**^ community; **β0** is the intercept-the effect on the probability of households’ receiving cash or food from PSNP in the absence of influence of independent variables; and **β’s** is the fixed coefficient.

The variance and intraclass correlation coefficient (ICC) for each model were calculated to determine whether the data had a dependency and correlation nature. ICC was calculated based on the following equation [37]:

$$\mathrm{ICC}=\;f^2/(f^2+\;\mathrm\pi^2)$$

where ƒ is an estimated variance.

The data are dependent and correlated if the ICC value is greater than 0.25 [31]. Consequently, 62% of the ICC value confirmed that there were significant correlations between households receiving cash or food from PSNP (Table 3). Similarly, the variance indicated that there was a 66% significant variation in receiving cash or food from the PSNP that violates the equal probability sample allocation assumption. So, these all indicated that there was a data dependency and correlation. The log-likelihood ratio (LLR) was used for model comparison. To solve the dependency and correlation within records, the model with the highest LLR value was chosen as the best-fit model [32]. As a result, model D was chosen as the best-fit model due to its LLR score’s highest value (-2598.4) as compared to other models (Table 3). In multilevel mixed effect logistic regression analysis, a p-value less than 5% with a 95% CI was used to identify factors associated with receiving cash or food from PSNP among households in Ethiopia.

### Ethics approval and consent to participant

This study was based on a secondary data source that is publicly available from the Measure DHS Program website (https://dhsprogram.com). Therefore, ethical approval and consents from study participants were not necessary for this study.

A request was sent to the Measure DHS program to get permission to access and use the 2019 EMDHS data from (https://dhsprogram.com/Date/terms-of-use.cfm). Then we got permission for data access and use.

## Results

### Sociodemographic characteristics of the study

A total of 8595 weighted samples were used in this study. Around one-fourth (24.1%) of households were in the Amhara region, whereas 37.0% of households were from the Oromia region. The majority (69.2%) of households were rural residents. 44.5% of household heads were older than 44 years, and 77.9% of households were male. 44.6% of the households were wealthy. Households that had no television or radio were 83.1% and 72.2%, respectively (Table 1).

**Table 1:** Sociodemographic characteristics of the study participants using the 2019 EMDHS dataset

Variable	Category	Frequency (n)	Percent (%)
Place of residency	Urban	2647	30.8
	Rural	5948	69.2
Region	Tigray	582	6.8
	Afar	87	1.0
	Amhara	2081	24.2
	Oromia	3184	37.0
	Somali	417	4.9
	Benishangul-Gumuz	93	1.1
	SNNPR	1661	19.3
	Gambela	35	.4
	Harari	25	.3
	Addis Ababa	375	4.4
	Dire Dawa	55	.6
Age of household head	15–24 years	650	7.6
	25–34 years	2038	23.7
	35–44 years	2084	24.2
	> 44 years	3824	44.5
Sex of household head	Male	6697	77.9
	Female	1899	22.1
Households’ wealth status	Poor	3104	36.1
	Middle	1663	19.3
	Rich	3829	44.6
Households have television	No	7147	83.1
	Yes	1448	16.9
Households have radio	No	6203	72.2
	Yes	2393	27.8

### The spatial distribution of households cash or food received from the PSNP

**Fig. 1:**
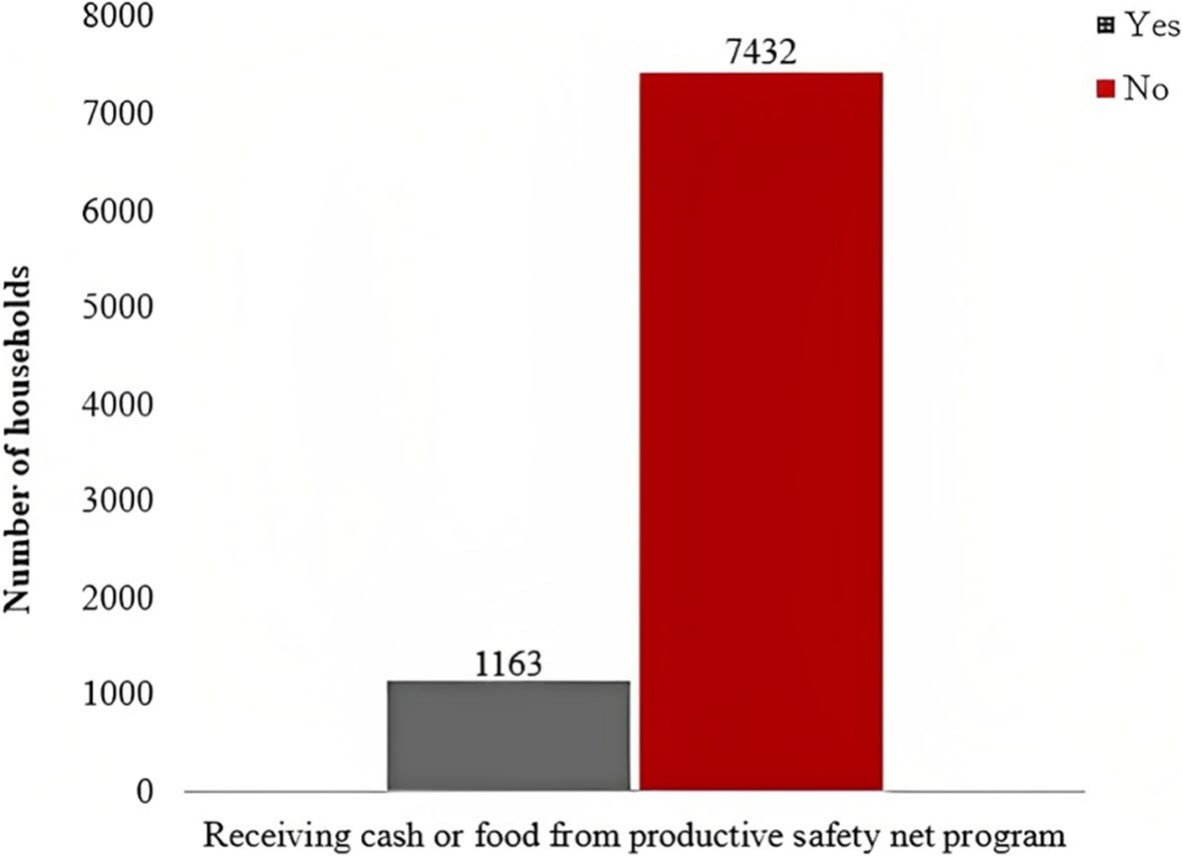
Cash or food received from PSNP among households in Ethiopia using the 2019 EMDHS dataset

Overall, nearly one-seventh (13.5% (95% CI: 12.81–14.27%)) of the household-level beneficiaries received cash or food from the PSNP in Ethiopia. Receiving cash, or food from the PSNP was good in Addis Ababa, the SNNPR, Amhara, and Oromia regional states of Ethiopia. However, Benishangul Gumuz, Dire Dawa, Harari, Afar, and Gambela regions had less access to cash or food from the PSNP ( Fig. 1).

**Fig. 2:**
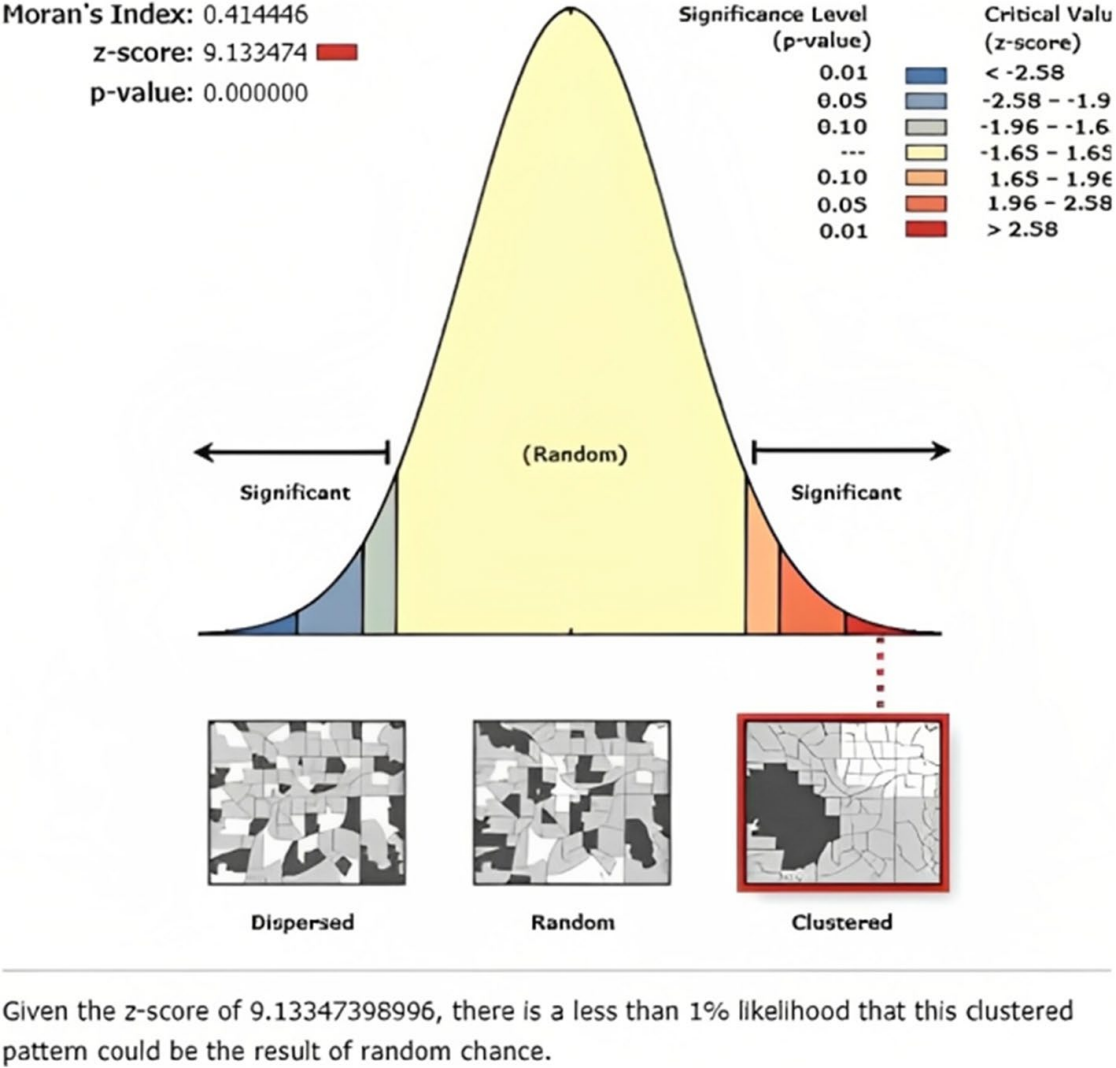
Spatial autocorrelation report of cash or food received from the PSNP among households using the 2019 EMDHS dataset

**Fig. 3:**
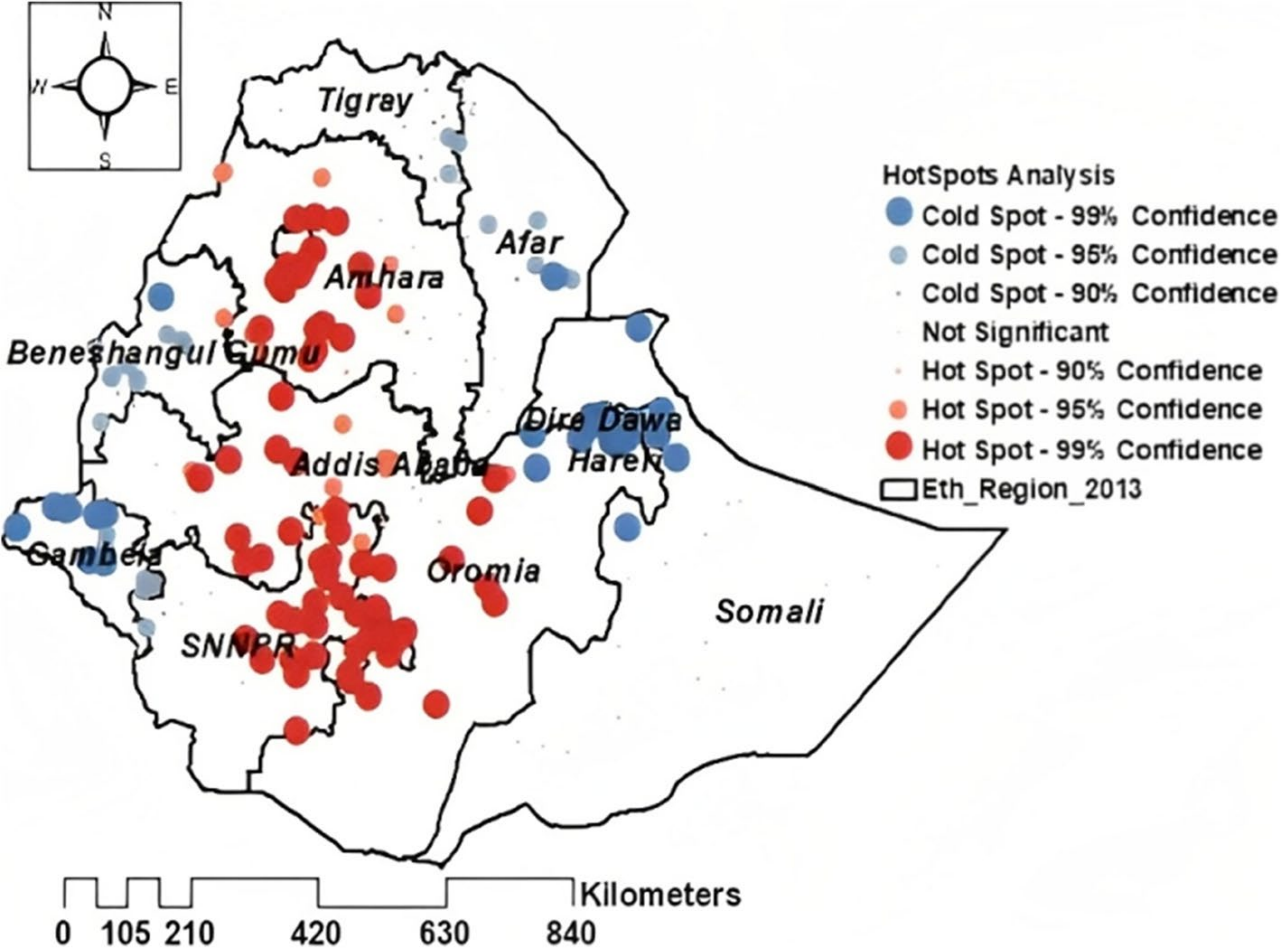
Hotspot analysis of cash or food received from the PSNP among households using the 2019 EMDHS dataset

The spatial distribution of households' cash or food received from the PSNP was significantly clustered across the regions of Ethiopia (Global Moran’s I = 0.414446, *P*-value = 0.00000) within 155,149.8 m of threshold distance (Figs. 2, and 3).

### Spatial SaTScan analysis

**Fig. 4:**
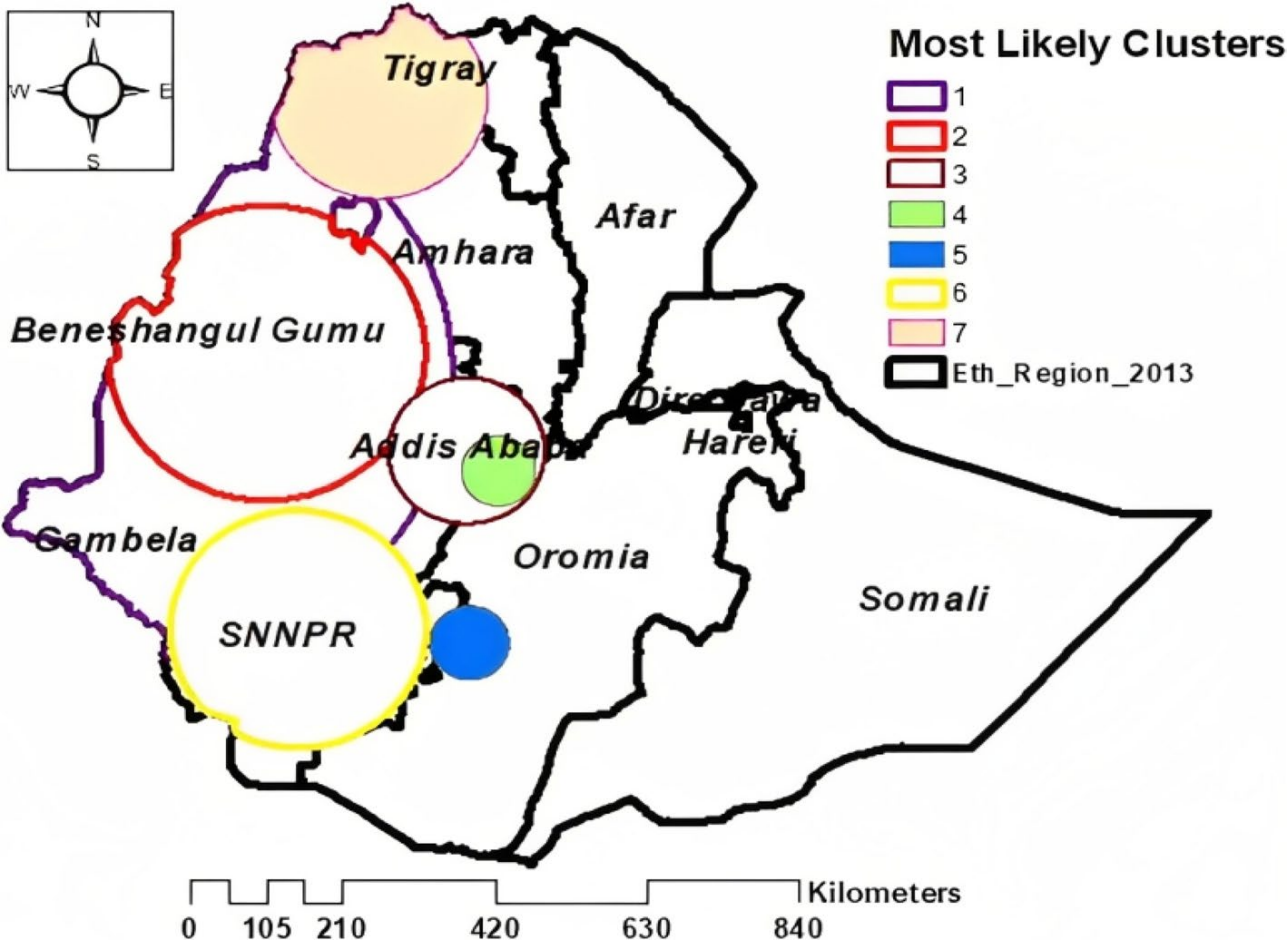
SaTScan analysis of cash or food received from the PSNP among households in Ethiopia using the 2019 EMDHS dataset

A total of 234 significant clusters for PSNP among households were identified. Of the 234 clusters, 97, 48, and 31 were primary, secondary, and tertiary clusters, respectively. The primary clusters were located at 9.921716 N, 34.697854 E within a 421.15 km radius in the Gambela, Benishangul Gumuz, western Amhara, and Oromia regions of Ethiopia. The secondary clusters were located at 10.219055 N, 34.209810 E within a 421.88 km radius in the Benishangul Gumuz, the western part of Amhara, and the northern part of Gambela regions of Ethiopia. Significant clusters were identified in the west, south, and northwest of Ethiopia. Households in the primary, secondary, and tertiary clusters were 23%, 19%, and 15% more likely to receive cash or food from the PSNP than households outside the window respectively (Table 2, Fig. 4).

**Table 2:** Significant clusters for the spatial Sat Scan analysis of cash or food received from the PSNP among households in Ethiopia using the 2019 ENDHS dataset

Types	Detected cluster	Coordinate/ Radius	Population	Case	RR	LLR	*P*-value
Primary	154, 153, 155, 152, 147, 86, 151, 157, 156, 150, 149, 170, 146, 160, 118, 169, 158, 161, 168, 167, 159, 166, 92, 164, 164, 218, 2017, 211, 120, 208, 209, 163, 230, 229, 217, 94, 220, 93, 162, 212, 165, 213, 80, 77, 79, 214, 206, 219, 119, 98, 194, 225, 226, 97, 221, 227, 228, 222, 223, 210, 224, 87, 75, 53, 52, 72, 96, 201, 74, 195, 76, 201, 74, 195, 76, 91, 200, 54, 81, 70, 95, 215, 59, 99, 71, 73, 85, 174, 112, 55, 57, 216, 84, 171, 176, 82, 179	9.921716N, 34.697854E/ 421.15 km	3104	1700	1.23	388.8	< 0.001
Secondary	164,166, 148, 163, 167, 161, 168, 77, 158, 169, 80, 162, 79, 160, 119, 165, 150, 93, 156, 149, 86, 89, 52, 159, 92, 72,155, 120, 53, 154, 75, 153,76, 118, 157,151, 152, 147, 70,87, 74, 81,54,170,146,71,73, 59	10.219055N, 36.209810E/ 217.88 km	1702	1685	1.19	209.3	< 0.001
Tertiary	274, 277, 279, 275, 276, 270, 278, 260, 280, 261, 264, 273, 263, 259, 267, 265, 258, 271, 257, 262, 266, 256, 272, 268, 269, 101, 90, 175, 112, 99, 171	8.915417N, 38.733276E/ 107.76 km	953	931	1.15	80.45	< 0.001
Fourth	101, 90, 280, 278, 279, 272, 271, 277	8.651588N, 39.118340E/ 51.09 km	337	336	1.16	48.86	< 0.001
Fifth	113, 183, 186, 182, 181, 117, 115	6.362562N, 38.759281E/ 54.77 km	383	370	1.12	23.65	< 0.001
Sixth	196,173,192,204,198,191,195,199, 197,190,96, 201, 91, 189, 200, 194,97,223,202, 215, 210, 180, 222, 224,227, 179, 178, 221, 216	6.540286N, 36.627468E/ 175.77 km	1083	994	1.07	91.8	< 0.001
Seventh	23, 9, 8, 21, 56, 1, 7, 6, 13, 4, 12, 84, 82, 2	7.648661N, 37.601189E/ 149.03 km	421	399	1.10	94.80	< 0.001

### Interpolation of cash or food received from the PSNP among households in Ethiopia

**Fig. 5:**
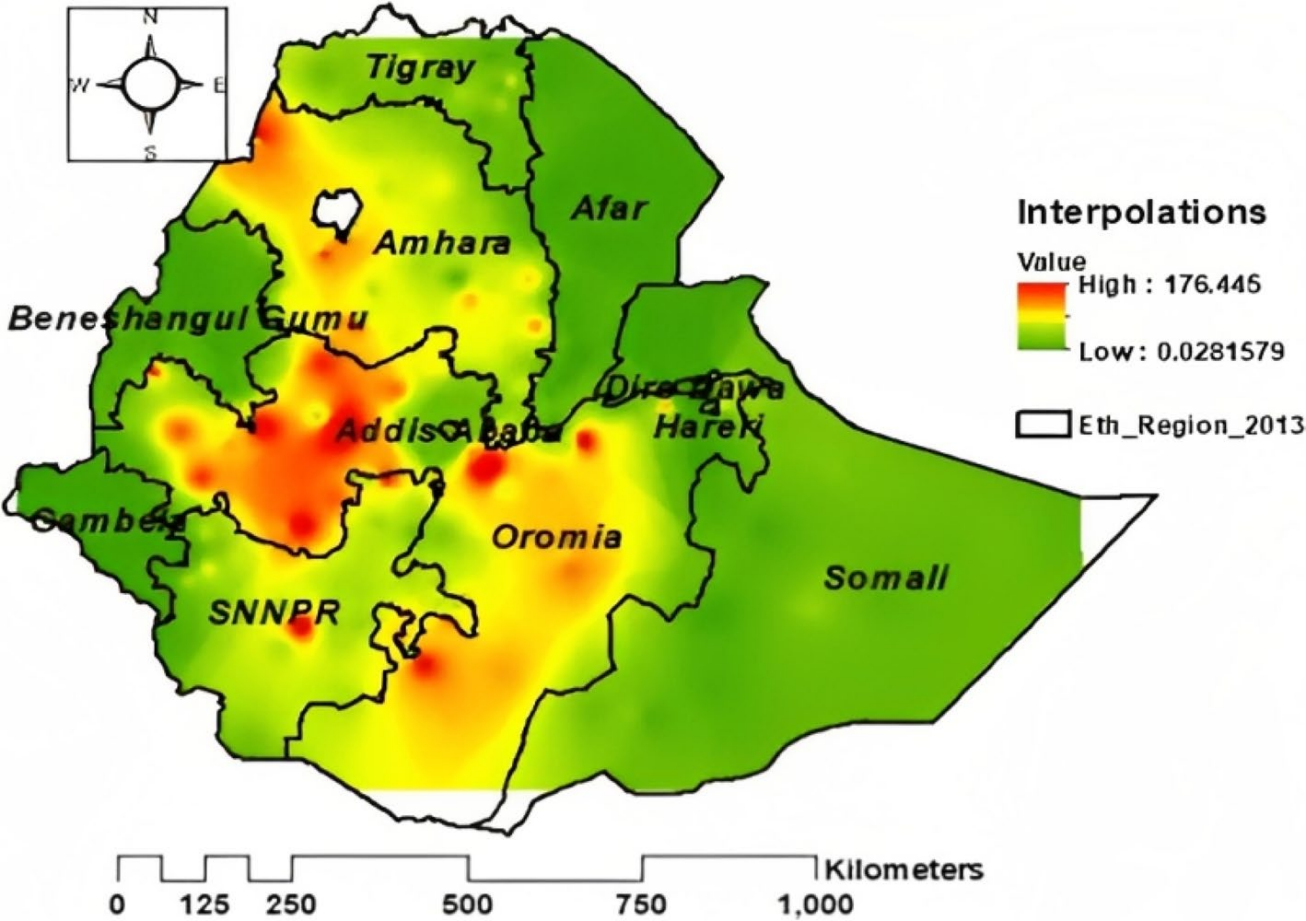
Interpolation of cash or food received from the PSNP among households in Ethiopia using the 2019 EMDHS dataset

The Gaussian Kriging interpolation method was employed. The interpolation result indicated that households in the SNNPR, Oromia, and Amhara regions were more likely to receive cash or food from the PSNP. However, low access to cash or food from the PSNP among households occurred in the remaining regions of Ethiopia (Fig. 5).

### Factors associated with cash or food received from the PSNP among households in Ethiopia

In multivariate multilevel mixed effect logistic regression analysis, age and sex of the household head, wealth, enrollment in the CBHI, region, and place of residency were statistically significant factors associated with cash or food received from the PSNP among households.

Poor wealth status households were 1.9 (AOR: 1.91, 95% CI: 1.52, 2.39) times more odds to receive cash or food from the PSNP than rich wealth status households. Female-headed households were 1.5 (AOR: 1.51, 95% CI: 1.27,1.79) times more likely to receive cash or food from the PSNP than male-headed households.

Household heads who were under 25–34, 35–44, and greater than 34 years of age were 1.4 (AOR:1.43, 95% CI: 1.02, 2.00), 2.4 (AOR: 2.41, 95% CI: 1.72, 3.37), and 2.5 (AOR: 2.54, 95% CI: 1.83, 3.51) times more likely to receive cash or food from the PSNP than 15–24 years of age. Rural households were 2.2 (AOR: 2.18, 95% CI: 1.21,3.94) times more likely to receive cash or food from the PSNP than urban households. Households in the Amhara and Oromia regions were 14% (AOR: 0.14, 95% CI: 0.06, 0.39) and 36% (AOR: 0.36, 95% CI:0.12, 0.91) more likely to receive cash or food from the PSNP, respectively. Households enrolled in the CBHI were 3.3 (AOR: 3.34, 95% CI:2.69,4.16) times more likely to receive cash or food from the PSNP than households unenrolled in the CBHI (Table 3).

**Table 3:** Multilevel mixed-effect logistic regression analysis of factors associated with cash, or food received from the PSNP among households in Ethiopia

Variables	Category	Receiving cash/ food		Model A	Model B	Model C	Model D
					AOR (95% CI)	AOR (95% CI)	AOR (95% CI)
		No	Yes				
Media exposure	Yes	2889	282		0.51(0.34, 0.70)^b^	-	0.47(0.34, 0.67)
	No	4543	881		1		1
Wealth	Poor	2457	646		2.02 (1.61, 2.52)^b^	-	1.91(1.52, 2.39)^a^
	Middle	1448	215		2.70 (1.55, 3.90)	-	2.68 (1.54, 3.90)
	Rich	3527	302		1		1
Household head sex	Female	1546	352		1.55 (1.30, 1.84)^b^	-	1.51(1.27,1.79)^a^
	Male	5886	811		1	-	1
Age	25–34 years	1834	198	-	1.45 (1.00, 1.95)^b^	-	1.43 (1.02, 2.00)
	35–44 years	1767	317	-	2.43 (1.33, 3.26)^b^	-	2.41 (1.72, 3.37)^a^
	> 44 years	3217	607	-	2.63 (1.76, 3.36)^b^	-	2.54 (1.83, 3.51)^a^
	15–24	609	41	-	1		1
Region	Afar	41	46		-	14.96 (5.6, 40.4)^b^	13 (5.07,36)
	Amhara	1871	210		-	0.15 (0.06, 0.40)^b^	0.14 (0.06, 0.39)^a^
	Oromia	2793	390		-	0.37 (0.14, .96)^b^	.36 (0.12, 0.91)^a^
	Somali	319	98		-	2.28 (0.84, 6.20)	
	Benishangul	91	2		-	0.07 (0.02, 0.22)^b^	0.06 (0.02, 0.20)
	SNNPR	1393	268		-	0.52 (2.1, 1.33)	
	Gambela	33	2		-	0.42 (0.15, 1.17)	
	Harari	21	4		-	0.93 (0.33, 2.67)	
	Addis Ababa	354	22		-	0.74 (0.23, 2.36)	
	Dire Dawa	45	10		-	2.35 (0.84, 6.62)	
	Tigray	472	110			1	1
Residency	Rural	5025	923	-	-	3.8 (2.15, 6.94)^b^	2.18 (1.21,3.94)^a^
	Urban	2407	240			1	1
Enrollment in CBHI	Yes	1922	495	-	-	3.24 (2.62,4.01^b^	3.34 (2.69,4.16)^a^
	No	5510	669	-	-	1	1
LLR				-2790	-2658.97	-2732.05	-2598.4
AIC				5584	5337.95	5490.1	5238.8
Variance				0.66	0.60	0.38	0.34
ICC				0.62	0.59	0.47	0.44

b Significant at model B and C, a Significant at model D, 1 Reference

## Discussion

In this study, the 2019 Ethiopian Mini Demographic and Health Survey dataset was used as a data source, and a total of 8595 weighted sample households were included for analysis. A multilevel mixed-effect logistic regression model was applied to carry out the statistical analysis and to overcome the dependency between records. According to spatial analysis reports, cash or food received from the PSNP was significantly clustered in Ethiopia, and it was high in the SNNPR, Amhara, and Oromia regions. Purely spatial Bernoulli-based model scan statistics were used to locate statistically significant clusters. As a result, a total of 234 significant clusters were identified. Households in the primary, secondary, and tertiary clusters were 23%, 19%, and 15% more likely to receive cash or food from the PSNP than households outside the window. Unsampled areas were predicted based on the sampled area by using Gaussian Kriging interpolation techniques. Therefore, households except in the SNNPR, Oromia, and Amhara regions were less likely to receive cash or food from the PSNP.

Generally, only 13.5% of the households received cash or food from the PSNP in Ethiopia. This finding was lower than a study done in Ethiopia which stated that 29.1% of the households benefited from the program [13]. Plus, the finding was low in comparison to the program's goals and objectives of supporting 7.6 million people [13, 38] and targeting 5 and 8.3 million chronically food-insecure households in Ethiopia in the first and second phases, respectively [17, 19]. However, the existing evidence was supported by a study that states 77.1% of households are suffering from food insecurity [4]. This might be due to the program implementation process in the selected regions [39], and the program may deliver short-term nutritional benefits [40]. The criteria used to include and exclude households might be wrongly designed. So, the PSNP might have neglected eligible beneficiaries in the program [41], and households’ graduation from the PSNP might not be satisfactory [23]. Additionally, the PSNP might be more likely to work on natural resource (water and soil) reservations, the construction of roads, pumping water, schools, and clinics that can serve the whole community. Furthermore, the PSNP could work on agricultural production, technology adoption, and improved disaster and climate risk management [21].

In the multilevel mixed effect logistic regression analysis, independent variables such as age, sex, wealth status, enrollment in the CBHI, region, and place of residency were factors associated with cash or food received from the PSNP among households.

Poor households were 1.9 times more likely to receive cash or food from the PSNP than rich households. This finding was supported by evidence about conditional cash transfer programs [14]. This might be because households in low-income countries are more likely to face food insecurity than those with high household incomes [4]. Furthermore, because the PSNP’s primary goal is to provide social protection and poverty reduction, asset building for poor households [42], and ensuring opportunities for poor households to use their assets than wealthy households. Food and cash transfers may typically target chronically poor households to reduce their vulnerability and risk of falling back into poverty [14].

Female-headed households were 1.5 times more likely to receive cash or food from the PSNP than male-headed households. This finding was supported by studies done in Ethiopia [23, 43]. Additionally, the evidence was supported by a report that states that social protection programs and public works mainly target women, with a strong focus on addressing the poverty of female-headed households and encouraging women ‘s participation in public works activities [44]. This is probably due to women’s access to and control over income being key factors in ensuring household food security [45]. This means that women’s access to food is closely connected to households’ food access [11]. However, men might be responsible for income-generating activities and big nonfood purchases [46, 47]. The program may aim to empower and support nutritionally vulnerable women [48]. Moreover, the social and economic risks might be significantly higher for women as compared with men. Women, for example, may have less education and credit, a smaller social network, and are more likely to be violated for sexual intercourse. Due to all these reasons, the PSNP might be more concerned about female-headed households [44].

Household heads who were under 25–34, 35–44, and greater than 34 years of age were 1.4, 2.4, and 2.5 times more likely to receive cash or food from the PSNP than those who were 15–24 years of age. This finding was supported by a study done in southeast Ethiopia [21], which reported that as the age of the beneficiary increases, the beneficiaries who graduated with a higher level of PSNP are more likely to receive benefits from PSNP. As the beneficiary’s age increases, their understanding of the program’s objectives and targets might also increase. The current evidence was also in line with a study that states that the minimum age of beneficiaries is 18 and above [13].

Households in the Amhara and Oromia regions were 14% and 36% more likely to receive cash or food from the PSNP in Ethiopia, respectively. This finding was supported by a study [49], and a report that states the PSNP is ineffective in terms of food security or child dietary diversity [17]. This might be due to the program’s implementation in selected regions [39], and the relatively low cash payment for the beneficiaries in the Amhara and Oromia regions [11]. Plus, beneficiaries might not receive the benefits on time [49], less attention and unsustainable management support for the program members, and they might use the received cash for productivity [50], which would benefit them further upon graduation, and households in Amhara and Oromia regions are more likely to leave the program [20]. Moreover, in these regions, local program task forces at the woreda, kebele, and community levels might not be established and functional, and the beneficiaries of the program might be higher than the resources of the safety net can cover [49].

Rural households were 2.2 times more likely to receive cash or food from the PSNP than urban households. This might be due to programs that aim to focus on rural households for increasing infrastructure, health service access, educational provision, agriculture and livestock production [51, 52]. Food insecurity in households can be solved through sustainable agricultural productivity and development. Therefore, the programs may invest more in the rural side of the community to increase agricultural productivity [16].

Households enrolled in CBHI were 3.3 times more likely to receive cash or food from the PSNP than households unenrolled in CBHI. This finding was similar to studies done about food insecurity in different regions of Ethiopia [4, 53, 54]. This could be households with access to debit and credit; participation in the insurance system can provide them with opportunities in various income-generating activities, which could improve their financial capacity to deal with the food shortage situation by stabilizing their food purchasing power [43, 55]. Furthermore, the Ethiopian government implements CBHIS in food-insecure rural districts to reduce the financial costs of illness. Being a member of CBHIS may be a sign of being a member of social protection programs that potentially target chronically food-insecure households to reduce their poverty level. So, interlinkages between the programs might exist. Such an approach is potentially promising in terms of helping the most vulnerable households deal with multiple shocks while at the same time increasing demand for insurance and protection [24, 56].

## Conclusions and recommendations

This study reports that eligible households have low access to cash or food from the PSNP in Ethiopia. Low access to cash or food from the PSNP among eligible households was detected in all regions of the country except in the SNNPR, Amhara, and Oromia regions. Independent variables such as household heads’ age and sex, wealth status of the household, region, place of residency, and enrollment in CBHS were statistically significant factors for receiving cash or food from the PSNP in Ethiopia. So, programmers would encourage poor and rural households to receive cash or food from the PSNP, and encourage them to be more productive with the benefits they receive from the PSNP. Programmers and stakeholders encouraged to give priority attention to the hotspot areas for the households that had not received cash or food from PSNP, especially poor households. The programmers recommended to redesign better strategies that would shape cultural patterns and social norms in the community, which may typically affect the food intake of households from PSNP. Moreover, high attention from programmers and stakeholders would required to create awareness among eligible households to use the benefits for production instead of consumption and avoid intra-household disparity in food distribution.

### Strengths and limitations of the study

This study was based on nationally representative data and locates households that receive cash or food from the PSNP spatially. This study used a multilevel mixed-effect logistic regression model to smooth the level of dependency and correlations that exist between records. So, the findings of the study had representativeness and would serve as input for decision-makers. As a limitation, since the data were collected retrospectively, and so recall bias might exist. Due to the coordinate files were not collected in the four-corner directions of Ethiopia, study participants in these areas may be excluded. In the SaTScan analysis, the result is presented in circular shapes, and results in irregularly shaped clusters may be clipped/ excluded.

## Data Availability

The dataset used for analysis is available on the Measure DHS program (http://dhsprogram.com) website. All the data generated and analyzed during this study are included, in the form of maps, tables, and texts, in this article.

## Abbreviations

CBHIS: Community-Based Health Insurance Scheme
CI: Confidence Interval
CSA: Central Statistical Agency
DHS: Demographic and Health Survey
EAs: Enumeration Areas
EDHS: Ethiopian Demographic and Health Survey
EMDHS: Ethiopia Mini Demography and Health Survey
EPHC: Ethiopian Population and Housing Census
EPHI: Ethiopian Public Health Institute
GTP: Growth and Transformation Plan
PSNP: Productive Safety Net Program
RR: Relative Risk
LLR: Loglikelihood Ratio
SDG: Sustainable Development Goals
SNNPR: South Nations and Nationality and Peoples of the region
STATA: Statistical software for data science

## References

[1] Chowdhury MRK (2016). Low maternal education and socio-economic status were associated with household food insecurity in children under five with diarrhoea in Bangladesh. Acta Paediatr.

[2] Smith MD, Rabbitt MP, Coleman-Jensen A (2017). Who are the world’s food insecure? New evidence from the Food and Agriculture Organization’s food insecurity experience scale. World Dev.

[3] Organization, W.H., The state of food security and nutrition in the world 2019: safeguarding against economic slowdowns and downturns. Vol. 2019. 2019: Food & Agriculture Org.

[4] Derso A (2021). Food insecurity status and determinants among urban productive safety net program beneficiary households in addis ababa, Ethiopia. PLoS ONE.

[5] Keeley, B., C. Little, and E. Zuehlke, The State of the World's Children 2019: Children, Food and Nutrition--Growing Well in a Changing World. UNICEF, 2019.

[6] Fanzo, J., et al., 2018 Global Nutrition Report. 2019.

[7] Poverty rate in Ethiopia, 2023. https://www.macrotrends.net/countries/ETH/ethiopia/poverty-rate.

[8] Unicef and W. WHO, Levels and trends in child malnutrition: key findings of the (2019). edition of the Joint Child Malnutrition Estimates.

[9] Ethiopian Public Health Institute (EPHI) [Ethiopia] and ICF. Rockville, M., USA: EPHI and ICF., Ethiopia Mini Demographic and Health Survey 2019: Final Report. 2021:Accessed from. https://dhsprogram.com/publications/publication-FR363-DHS-Final-Reports.cfm.

[10] Devereux, S. and A. Teshome, From safety nets to social protection: options for direct support beneficiaries. Food security, safety nets and social protection in Ethiopia, 2013: p. 67.

[11] Sabates-Wheeler R, Devereux S (2010). Cash transfers and high food prices: Explaining outcomes on Ethiopia’s Productive Safety Net Programme. Food Policy.

[12] Devereux, S., et al., Ethiopia’s Productive Safety Net Programme (PSNP): 2008 Assessment Report. IDS, Sussex, 2008.

[13] Porter C, Goyal R (2016). Social protection for all ages? Impacts of Ethiopia’s Productive Safety Net Program on child nutrition. Soc Sci Med.

[14] De Janvry, A., et al., Uninsured risk and asset protection: Can conditional cash transfer programs serve as safety nets? The World Bank, Social Protection Working Paper, 2006.

[15] Group, W.B., Ethiopia’s Productive Safety Net Program (PSNP) Integrating Disaster And Climate Risk Management. 2013, Available from: https://documents1.worldbank.org/curated/en/893931468321850632/pdf/806220WP0P12680Box0379812B00PUBLIC0.pdf.

[16] Fadong L (2018). Understanding agriculture production and food security in Ethiopia from the perspective of China. J Resour Ecology.

[17] Bahru BA (2020). Impact of Ethiopia's productive safety net program on household food security and child nutrition: a marginal structural modeling approach. SSM-population health.

[18] Hailu AG, Amare ZY (2022). Impact of productive safety net program on food security of beneficiary households in western Ethiopia: A matching estimator approach. PLoS ONE.

[19] Sharp, K., T. Brown, and A. Teshome, Targeting Ethiopia’s productive safety net programme (PSNP). London and Bristol, UK: Overseas Development Institute and the IDL Group, 2006.

[20] Berhane G (2011). The impact of Ethiopia’s productive safety nets and household asset building programme: 2006–2010.

[21] Welteji D, Mohammed K, Hussein K (2017). The contribution of Productive Safety Net Program for food security of the rural households in the case of Bale Zone. Southeast Ethiop Agric Food Secur.

[22] Tadesse, T. and T. Gebremedhin Zeleke, The impact of the productive safety net program (PSNP) on food security and asset accumulation of rural households’: evidence from Gedeo zone, Southern Ethiopia. Cogent Economics & Finance, 2022. 10(1): p. 2087285.

[23] Hayalu, G., Assessment of Factors Affecting Household Level Graduation from Productive Safety Net Program (PSNP): Evidence from Emba-Alaje District Southern Tigray, Northern Ethiopia. 2014, Mekelle University.

[24] Shigute Z (2017). Uptake of health insurance and the productive safety net program in rural Ethiopia. Soc Sci Med.

[25] Devereux, S., et al., Ethiopia’s productive safety net programme (PSNP). Trends in PSNP transfers within targeted households. Final report. Sussex, UK: Institute of Development Studies and Indak International, 2006.

[26] Kebede SA (2020). Spatial distribution and associated factors of health insurance coverage in Ethiopia: further analysis of Ethiopia demographic and health survey, 2016. Arch Public Health.

[27] AGRICULTURE AND FOOD SECURITY. 2022, Accessed from: https://www.usaid.gov/ethiopia/agriculture-and-food-security.

[28] Demsash AW (2022). Spatial distribution of vitamin A rich foods intake and associated factors among children aged 6–23 months in Ethiopia: spatial and multilevel analysis of 2019 Ethiopian mini demographic and health survey. BMC Nutrition.

[29] Levy, P.S. and S. Lemeshow, Sampling of populations: methods and applications. 2013: John Wiley & Sons.

[30] Anselin L, Getis A (1992). Spatial statistical analysis and geographic information systems. Ann Reg Sci.

[31] O'Sullivan D (2003). Geographically weighted regression: the analysis of spatially varying relationships. Geogr Anal.

[32] Chaikaew N, Tripathi NK, Souris M (2009). Exploring spatial patterns and hotspots of diarrhea in Chiang Mai. Thailand Int J Health Geogr.

[33] Kulldorff, M., Information Management Services, Inc. SaTScan™ version 4.0: software for the spatial and space-time scan statistics, 2004. 2009.

[34] Kulldorff M (1997). A spatial scan statistic. Commun Stat-Theory methods.

[35] Alemu K (2014). Spatiotemporal clusters of malaria cases at village level, northwest Ethiopia. Malar J.

[36] Hox, J.J., M. Moerbeek, and R. Van de Schoot, Multilevel analysis: Techniques and applications. 2017: Routledge.

[37] Adane B (2020). Factors associated with postnatal care utilization among postpartum women in Ethiopia: a multi-level analysis of the 2016 Ethiopia demographic and health survey. Arch Public Health.

[38] Holmemo, C., Project Information Document (Concept Stage)-ET Productive Safety Nets IV (PSNP 4)-P146883. 2014.

[39] Gilligan DO, Hoddinott J, Taffesse AS (2009). The impact of Ethiopia's Productive Safety Net Programme and its linkages. J Dev Stud.

[40] Debela BL, Shively G, Holden ST (2015). Does Ethiopia’s Productive Safety Net Program improve child nutrition?. Food Secur.

[41] Ethiopia’s Productive Safety Net Programme: Power, Politics and Practice. Accessed from: https://www.ennonline.net/fex/53/ethiopiassafetynetprogramme.

[42] Berhane, G., J.F. Hoddinott, and N. Kumar, The impact of Ethiopia’s productive safety net programme on the nutritional status of children: 2008–2012. Vol. 1604. 2017: Intl Food Policy Res Inst.

[43] Hassen K, Zinab B, Belachew T (2016). Gender and education as predictors of food insecurity among coffee farming households of the Jimma zone. Southwest Ethiop BMC Nutr.

[44] Jones N, Tafere Y, Woldehanna T (2010). Gendered risks, poverty and vulnerability in Ethiopia: To what extent is the Productive Safety Net Programme (PSNP) making a difference.

[45] FAO, The State of Food Insecurity in the World. 2014, Available from https://www.fao.org/3/i4030e/i4030e.pdf.

[46] Kennedy, E., Effects of sugarcane production in Southwestern Kenya on income and nutrition. Agricultural commercialization, economic development, and nutrition., 1994: p. 252–263.

[47] Purvis BM (1985). Family nutrition and women's activities in rural Africa. Food Nutr.

[48] Irenso, A.A. and G.E. Atomsa, Implications of Ethiopian Productive Safety Net Programme on household dietary diversity and women’s body mass index: a cross-sectional study. Food & Nutrition Research, 2018. 62. 10.29219/fnr.v62.1574 PMC6294832 30574045

[49] Negatu, W., Food security strategy and Productive Safety Net program in Ethiopia. Digest of Ethiopia’s national policies, strategies and programs, 2008: p. 1–22.

[50] Diriba, W., M. Kerime, and H. Kedir, The contribution of Productive Safety Net Program for food security of the rural households in the case of Bale Zone, Southeast Ethiopia. Agriculture and Food Security, 2017. 6(53).

[51] Gebresilassie YH (2014). The economic impact of productive safety net program on poverty: evidence from central zone of Tigray National Regional State, Ethiopia. Int J Innovative Res Dev.

[52] Wondim AK (2018). Impact of productive safety net program in rural community of Ethiopia: A review study. J Agric Ext Rural Dev.

[53] Gebru GW, Ichoku HE, Phil-Eze PO (2018). Determinants of livelihood diversification strategies in Eastern Tigray Region of Ethiopia. Agric Food Secur.

[54] Carswell, G., Livelihood diversification in southern Ethiopia. 2000.

[55] Gebre GG (2012). Determinants of food insecurity among households in Addis Ababa city, Ethiopia. Interdiscip Description Complex Syst.

[56] Hirvonen K, Bossuyt A, Pigois R (2021). Evidence from the Productive Safety Net Programme in Ethiopia: Complementarities between social protection and health policies. Development Policy Review.

